# The Emerging Facets of Non-Cancerous Warburg Effect

**DOI:** 10.3389/fendo.2017.00279

**Published:** 2017-10-23

**Authors:** Alyaa M. Abdel-Haleem, Nathan E. Lewis, Neema Jamshidi, Katsuhiko Mineta, Xin Gao, Takashi Gojobori

**Affiliations:** ^1^King Abdullah University of Science and Technology (KAUST), Computational Bioscience Research Centre (CBRC), Thuwal, Saudi Arabia; ^2^King Abdullah University of Science and Technology (KAUST), Biological and Environmental Sciences and Engineering (BESE) Division, Thuwal, Saudi Arabia; ^3^Novo Nordisk Foundation Center for Biosustainability, University of California San Diego School of Medicine, La Jolla, CA, United States; ^4^Department of Pediatrics, University of California, San Diego, La Jolla, CA, United States; ^5^Department of Bioengineering, University of California, San Diego, La Jolla, CA, United States; ^6^Institute of Engineering in Medicine, University of California, San Diego, La Jolla, CA, United States; ^7^Department of Radiological Sciences, University of California, Los Angeles, Los Angeles, CA, United States; ^8^King Abdullah University of Science and Technology (KAUST), Computer, Electrical and Mathematical Sciences and Engineering (CEMSE) Division, Thuwal, Saudi Arabia

**Keywords:** Warburg effect, cancer, immune cells, malaria, angiogenesis, pluripotency, rapid proliferation, constraint-based metabolic modeling

## Abstract

The Warburg effect (WE), or aerobic glycolysis, is commonly recognized as a hallmark of cancer and has been extensively studied for potential anti-cancer therapeutics development. Beyond cancer, the WE plays an important role in many other cell types involved in immunity, angiogenesis, pluripotency, and infection by pathogens (e.g., malaria). Here, we review the WE in non-cancerous context as a “hallmark of rapid proliferation.” We observe that the WE operates in rapidly dividing cells in normal and pathological states that are triggered by internal and external cues. Aerobic glycolysis is also the preferred metabolic program in the cases when robust transient responses are needed. We aim to draw attention to the potential of computational modeling approaches in systematic characterization of common metabolic features beyond the WE across physiological and pathological conditions. Identification of metabolic commonalities across various diseases may lead to successful repurposing of drugs and biomarkers.

## Introduction

While all cells need a source of energy to maintain homeostasis, proliferating cells require a substantial amount of nutrients to produce biosynthetic building blocks and macromolecules for the newly produced daughter cells (1). Both glycolysis and respiration through oxidative phosphorylation (OxPhos) can generate free energy in the form of adenosine-5′-triphosphate (ATP) (1). Most cells metabolize glucose to pyruvate *via* glycolysis, and under normoxic conditions, the generated pyruvate is further oxidized to CO_2_ in the mitochondria through OxPhos, generating up to 36 ATP molecules per glucose molecule. When oxygen becomes limiting, mitochondrial OxPhos is restricted and pyruvate is converted to lactate instead. However, it has been widely observed across different cell types that the latter can predominate when oxygen is plentiful (2). A common feature among cells exhibiting this phenomenon of aerobic glycolysis (3, 4) is “rapid proliferation.” Although it seems counterintuitive, most rapidly proliferating cells seem to rely on aerobic glycolysis despite the fact that it yields significantly less ATP/glucose compared to OxPhos (5, 6). Although different proposals have been put forward to rationalize the cell’s unique feature of using the Warburg effect (WE), it is still unclear whether aerobic glycolysis is “causal” or if it is just a phenotype of rapidly proliferating cells due to metabolites overflow (7).

**Figure 1:**
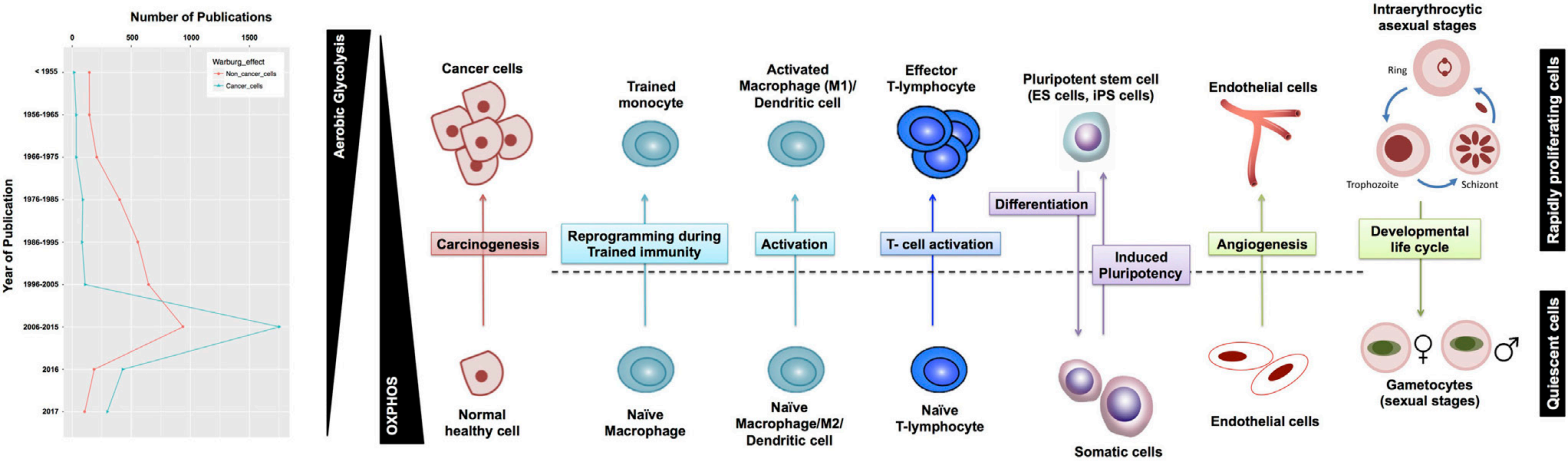
Warburg effect (WE) in cancerous and non-cancerous cells. **(A)** The frequency of publications on the WE in cancer cells has not witnessed a parallel surge in studies investigating the same phenomenon in non-cancerous context. **(B)** WE has been observed across a plethora of rapidly proliferating cells in both physiological and pathological contexts. T-cells shape is adopted from Ref. (9), the malaria life cycle stages image is adopted from Ref. (10), and the blood vessel icon is adopted from Ref. (11). ES, embryonic stem cells; iPS, induced pluripotent stem cells.

**Figure 2:**
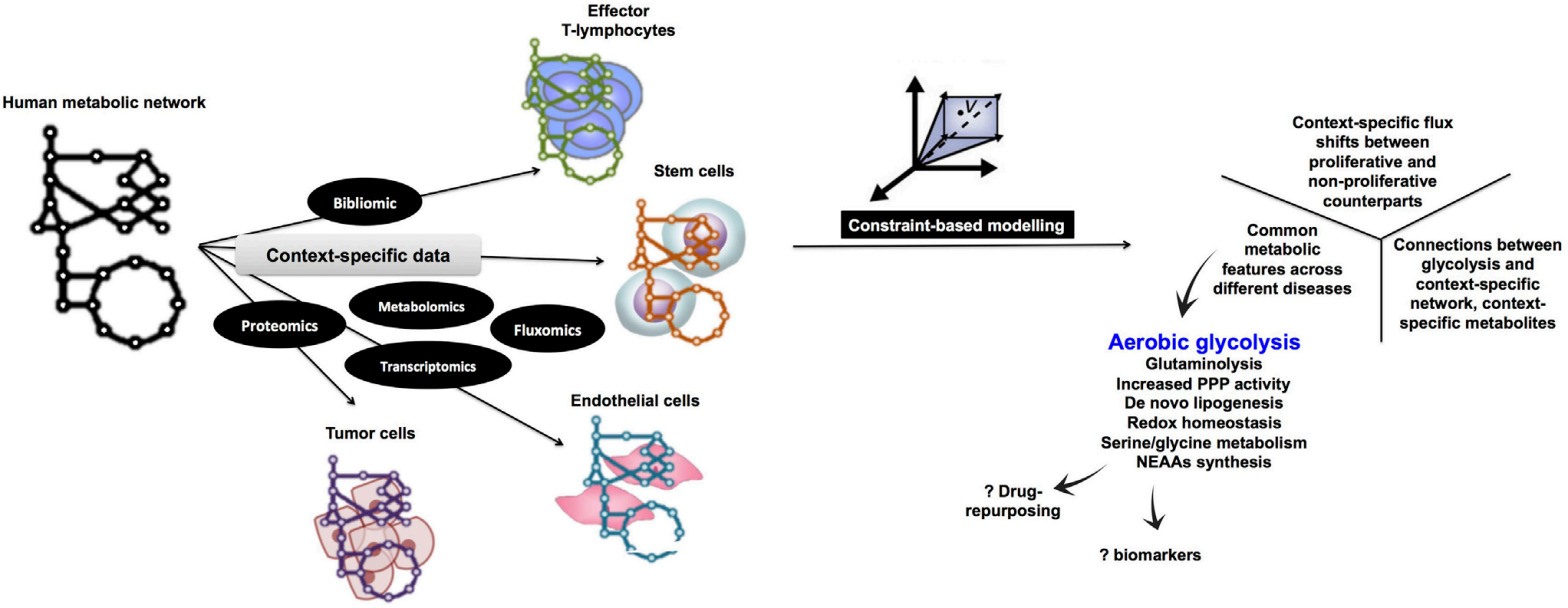
Proposed computational metabolic modeling approach to systematically identify common metabolic features within pathological conditions as well as across normal and disease states. Starting with a curated human metabolic network, high-throughput data specific for each normal tissue (e.g., proliferating endothelial cells and effector T lymphocytes) or disease (e.g., cancer, autoimmune diseases, and inflammation) will be used to develop the corresponding context-specific metabolic network which is amenable to simulations under the constraint-based modeling framework being subject to different levels of constraints. By assigning an appropriate objective function (e.g., biomass production), it is possible to enumerate metabolic processes that are tightly coupled to growth and proliferation. Eventually, since all context-specific models are developed under a uniform integrative framework, it is legitimate to cross compare metabolic networks potentially identifying common metabolic features (e.g., aerobic glycolysis). NEAAs, non-essential amino acids metabolism.

Although aerobic glycolysis is now an established hallmark of cancer (8), relatively fewer studies have investigated the WE in non-cancerous cells (Figure 1). Here, we discuss the role of aerobic glycolysis as a “hallmark of rapid proliferation” as part of cellular dysregulation (cancer, inflammation, and autoimmune diseases), physiologically regulated process (T-cell activation and angiogenesis), and pluripotency. Beyond mammalian cells, the WE has also been central to the developmental stages of rapidly proliferating parasites, such as Plasmodium and Toxoplasma. Furthermore, the use of aerobic glycolysis and the secretion of organic acids are common in most rapidly growing microbes (e.g., yeast and *E. coli*). We argue that cells adopt aerobic glycolysis in the cases where a rapid transient action is needed while respiration tends to support long-term constitutive (more stable) processes. Because the span of cells exhibiting the WE is wide, we propose that a systems biology approach based on constraint-based modeling (CBM) of metabolism (Figure 2) can be useful as a means of systematic characterization of common and distinct features of the WE across different diseases and cell types.

## We Plays a Role in Activation of Effector T-Helper Lymphocytes (Adaptive Immunity)

In order to perform their function in protecting the body against pathogens and allergens, naïve T-cells need to be activated. This involves rapid proliferation (clonal expansion) and differentiation of naïve T-cells into antigen-specific T-effectors (Teff) or T-regulatory cells that function to mediate or suppress immune responses, respectively (12–14). T-cell activation requires energy and metabolic precursors for macromolecular biosynthesis (15). The role of WE in T-cell activation has recently become more apparent, and efforts are underway to understand its role.

Upon stimulation, effector T-cells exhibit high levels of glucose uptake and glycolysis (16). In both cancer cells and activated effector T-cells, elevated expression of glucose transporter, particularly GLUT1, has been reported (12, 16, 17). However, it is unclear whether upregulation of GLUT1 happens as a prerequisite or consequence of T-cell activation as Teff was selectively increased in transgenic mice when Glut1 was overexpressed (18). Enhanced glucose uptake is also linked to increased expression and activity of glycolytic enzymes. In humans, chronically activated T-cells in allergic asthma patients produce high levels of lactate and overexpress pyruvate dehydrogenase kinase that inhibits pyruvate dehydrogenase, thus restricting the entrance of pyruvate into the mitochondrial TCA (19, 20). In addition to transporters and glycolytic enzymes, carcinogenesis shares many metabolic regulators with T-cell activation, including phosphoinositide 3-kinase [PI(3)K]/Akt, mammalian target of rapamycin complex (15, 16, 21), and Myc (15, 22) as well as the hypoxia-inducible factor-1α (HIF-1α) (2, 20). Altogether, activation of T-cells parallels carcinogenesis with respect to adopting glycolysis rather than OxPhos in presence of oxygen.

It is critical to highlight that T-cell activation is not accompanied merely by a switch from oxidative metabolism to glycolysis, but that both pathways coordinate to support bioenergetic demands (15). In fact, mitochondrial activity is enhanced in stimulated lymphocytes compared to their resting counterparts. The same observation has been extensively reviewed in the field of cancer biology, which contradicts the initial “WE” theory that cancer cells opt for aerobic glycolysis due to a defective mitochondria. Hence, the WE does not require a defective mitochondria to be the predominant metabolic program in rapidly dividing cells (1, 21, 23).

An important difference between glycolysis in T-cell activation and carcinogenesis is that carcinogenesis is a form of cellular dysregulation (24). By contrast, T-cell activation happens in both pathological (e.g., autoimmune diseases) and physiological contexts (25, 26). This further underscores that aerobic glycolysis is a feature that is tied to rapid proliferation independent of the context being normal or pathological state.

## Marcophages (Innate Immunity) Utilize Aerobic Glycolysis

Inflammatory cells, such as activated macrophages, upregulate glycolysis (2) in response to tissue injury or infection to cope with increased production of host defense factors, enhanced phagocytosis, and antigen presentation (2). In both activated inflammatory cells and cancer cells, glucose transporter GLUT1 is upregulated, lactate production is increased, and oxygen consumption by the mitochondria is decreased in the downstream events to PI(3)K-Akt1 signaling (2). There is also a marked switch from expression of the liver isoform of 2,6-phosphofructo-2-kinase/fructose-2,6-biphosphatase (encoded by PFKFB1) to the PFKFB3 isoform, the type of which is also commonly found in tumor cells. This leads to accumulation of fructose-2,6-bisphosphate (F2,6P_2_) as an allosteric activator of 6-phosphofructo-1-kinase (PFK1), and therefore, glycolysis takes place (2, 27, 28). In addition, hexokinase, the first enzyme involved in glycolysis as well as in the pentose phosphate pathway (PPP), is also upregulated in activated macrophages (29).

In contrast to pro-inflammatory M1 macrophages, anti-inflammatory M2 macrophages have higher rates of OxPhos and lower rates of glycolysis (2). Further, M2 macrophages have no detectable PFKFB3 and expressing PFKFB1 instead (2). In addition, the transcription factor HIF-1α and AMP-activated protein kinase play critical roles in regulating the metabolic alterations between inflammatory and anti-inflammatory responses (2, 30, 31). It is noteworthy that both M1 and M2 macrophages are highly active and can proliferate; nevertheless, the pro-inflammatory M1 relies on glycolysis while the anti-inflammatory and tissue-repair-promoting M2 relies on OxPhos. We thus hypothesize that OxPhos is more suited to long-term reparative roles (e.g., anti-inflammation), while aerobic glycolysis being suited for rapid, robust, and transient responses (e.g., inflammation). However, fitting this premise in the context of the behavior of quiescent adult stem cells, which opt for glycolysis to avoid senescence due to increased ROS load generated by OxPhos (5, 32), warrants further investigation.

In the pathological states discussed here, we observe that the WE is a phenotype of rapidly dividing cells irrespective of whether the context is triggered by an external or internal cue. For instance, autoimmune diseases might arise from somatic mutations in antigen receptors according to the “Clonal Selection Theory” (26). Similarly, cancer cells might arise due direct or indirect oncogenic mutations. In both cancer and autoimmune diseases, the trigger is an internal cue. However, in the case of physiological T-cell activation, external signals (cytokines in this case) mediate the appropriate immune response (2, 33).

Taken together, the WE is a hallmark of rapidly proliferative cells across wide spectrum of pathological and physiological processes that are triggered by either internal or external cues.

## Endothelial Cells (ECs) Utilize Aerobic Glycolysis During Angiogenesis

Blood vessels deliver oxygen and nutrients to all of the tissues and organs in the body. ECs and vascular smooth muscle cells are the two main cellular components of blood vessels. Consequently, these cells are involved in a variety of physiological processes as well as pathological dysfunctions, including atherosclerosis (34).

Angiogenesis relies on the proliferation and migration of ECs (35). Once the vessel is perfused, ECs become quiescent phalanx cells (27). Similar to other rapidly proliferating cells, ECs are exposed to sufficient oxygen for oxidative metabolism, yet they prefer aerobic glycolysis. Radioactive-tracer labeled substrates in EC monolayers showed that glycolytic flux in ECs was largely comparable to those in tumor cells but much higher than in various other healthy cells (27). Notably, glycolytic flux was more than 200-fold higher compared to glucose oxidation, fatty acid oxidation, and glutamine oxidation (27), while mitochondrial respiration was lower in ECs than in other oxidative cell types (27). In addition, glycolysis generated up to 85% of the total cellular ATP content (27) and regulated vessel sprouting (27). Overall, glycolysis is the predominant bioenergetic pathway for proliferating ECs.

Similar to other rapidly dividing cells discussed here, PFKFB3 is critical for EC proliferation where PFKFB3 silencing reduced vessel sprouting (27) while inhibition of respiration did not have a significant effect. Similar findings have been reported in cancer cells and M1 macrophages (36, 37), highlighting a potentially conserved critical role for PFKFB3 in aerobic glycolysis.

## Aerobic Glycolysis is a Metabolic Feature in Pluripotent Embryonic Stem Cells

In contrast to somatic cells and in analogy to rapidly proliferating cells, embryonic stem (ES) cells rely on glycolytic ATP generation regardless of oxygen availability (38–40). The reliance on glycolysis was suggested to be due to a low copy number of mitochondrial DNA (mtDNA) as well as low numbers of nascent mitochondria (38, 41). Differentiation increases mtDNA abundance and promotes mitochondrial biogenesis to form networks of elongated and cristae-rich mitochondria in support of competent oxidative metabolism (38, 42, 43).

High-resolution metabolomics showed that induced pluripotent stem (iPS) cells upregulate glycolytic enzymes and downregulate electron transport chain subunits enabling a switch that converts somatic oxidative metabolism into a glycolytic flux-dependent and mitochondria-independent state that underlies pluripotency induction (32, 38). To maintain high glycolytic rates, human embryonic stem cells as well as cells of the inner cell mass (which becomes the embryo proper) upregulate GLUT1, GLUT3, HK, and PFK1 (6, 32, 44–46) leading to increased lactate synthesis (44, 47). In iPS cells, the upregulation of glycolysis precedes the reactivation of pluripotency markers (48, 49) implicating that the glycolytic phenotype is more tied to rapid proliferation rather than pluripotency. Further, despite the low levels of oxygen consumption in undifferentiated ES, ATP synthesis is decoupled from oxygen consumption and depends on glycolysis instead, possibly consuming oxygen through the mitochondrial ETC (44). As ES progress toward differentiation, their glycolytic fluxes decrease dramatically while mitochondrial OxPhos fueled by glucose and fatty acids increases (44).

Elevated levels of PFKFB3 have been also reported in human embryonic kidney 293 cells (28) and cancer stem (CS) cells. However, iPS cells express a very low level of PFKFB3 while expression of PFK1 was comparable to that in CS cells. This indicates that PFK1 activation could be PFKFB3-independent in iPS cells (50). Taken together, PFKFB3 is upregulated in a wide spectrum of rapidly proliferating cells adopting Warburg metabolism.

## Malaria Adopts a Glycolytic Metabolic Program During Its Asexual Intraerythrocytic Life Cycle Stages

Malaria forms that are injected into human blood following an infected-mosquito bite, migrate to the liver, and then are released into the blood stream were they rapidly proliferate inside the red blood cells (RBCs), eventually causing malaria symptoms and pathology due to RBCs lysis (51). A small fraction (<1%) of these rapidly proliferative stages commit to sexual development and is responsible for transmitting infection to another mosquito vector (52). Because the malaria parasite encounters different metabolic niches across its developmental stages, its growth matches its nutritional requirements by rewiring its metabolic network [(53) and our unpublished work]. During the intraerythrocytic developmental stages, the asexual stages of the malaria parasite increase their glucose uptake by more than 10-fold (51) with 93% of their glucose uptake being converted into lactate (53), consistent with a high metabolic demand that is imposed by parasite division. This percentage drops to 80% in the non-proliferative/quiescent gametocyte stages (53). Hemoglobin digestion generates ROS and increases the redox burden, so that favoring aerobic glycolysis could be a means to minimize redox burden (compared to using OxPhos). Nevertheless, the asexual forms still rely on electron transport activity for regeneration of ubiquinone that is required as the electron acceptor for dihydroorotate dehydrogenase, an essential enzyme for pyrimidine biosynthesis (54). Knocking out the mitochondrial ATP synthase β-subunit gene that disrupted the parasite transmission cycle while only marginally reducing growth of the asexual rapidly proliferating stages, reflecting a higher essentiality of mitochondrial function in the non-rapidly proliferative mosquito stages (54). In another study, a genetic investigation of TCA metabolism across the malaria life cycle (55) showed that knocking out of six of the eight TCA cycle enzymes does not affect asexual growth while affecting life cycle progression in later stages. Collectively, these studies (51, 54, 55) show that the overall flux of pyruvate into the TCA cycle is low in the rapidly dividing sexual stages while aerobic glycolysis is more prominent. In contrast, elevated levels of the TCA cycle activity sustained by increased catabolism of pyruvate dominates in Plasmodium gametocytes.

In this context, the asexual forms of the malaria parasite converge metabolically with the rapidly proliferating counterparts of other cancerous and non-cancerous cells, as discussed here. Malaria is an obligate intracellular parasite and has lost several of its genome content leading to a reduced metabolic capacity compared to its host (56). The fact that the asexual rapidly proliferating forms of the parasite opt for the WE despite their reduced metabolic capacity compared to other rapidly proliferating eukaryotic cells implies that the synthesis of biosynthetic precursors does not necessarily come on top of the reasons for why cells preferentially undergo the WE.

## *In Silico* Metabolic Modeling Can Systematically Elucidate Common Metabolic Features Across Different Cell Types and Diseases

Constraint-based modeling (57, 58) uses genome-scale metabolic models (GEMs) as platforms for integrating and interpreting different levels of high-throughput data (59–62) (Figure 2). Under the constraints of substrate availability, mass conservation limits reaction products and their stoichiometry, while thermodynamics constrain reaction directionality. This information can be obtained from genome sequences and annotation (e.g., human genome annotation); organism-specific database (e.g., http://plasmodb.org for malaria) along with bibliomic data that support the presence of each metabolic functionality before being added to the metabolic reconstruction. A metabolic reconstruction is then converted to a stochiometric matrix (57, 58), based on the stochiometric coefficients of each reaction, which is amenable to computation and simulations. Data-driven network boundaries (e.g., uptake and secretion products) are then applied. Taken together, these constraints would define the allowable “solution space” to achieve a certain cellular objective (e.g., growth that can be simulated by biomass precursors production) (58, 63). Additional context-specific constraints (e.g., enzyme gene expression levels or metabolites concentration) would shrink the solution space leading to context-dependent predictions about the utilization of alternate pathways across the metabolic network.

Many CBM methods for analyzing genome-scale metabolic networks (64, 65) have been developed (58). Since certain enzymes are only active in specific cell types, COBRA methods can be used for tailoring a generic metabolic network (Figure 2) by integrating high-throughput data to extract a cell type or disease-specific metabolic model from a GEM.

To comprehensively identify common metabolic features across the range of rapidly proliferating cells we discuss here, we suggest a CBM-based workflow (Figure 2) to enable integration of different levels of data to model the widely variable types of rapidly proliferating cells. Because of the ability of CBM to predict gene essentiality by simulating single-gene knockouts (58, 63), GEMs can provide a means to address the systematic interactions between the different biological components of the WE along with elucidating how they influence the entire metabolic network. Furthermore, model-predicted knockout phenotypes that selectively inhibit growth of rapidly proliferating cell models but not their quiescent counterparts can be integrated in drug development pipelines to predict druggable targets (63, 66–68) as well as new drug combinations. Using a metabolite essentiality analysis (69), instead of gene-knockout experiments, biomarkers for identification of cells undergoing the WE can be predicted. The advantage of using metabolites prompt searching for structural analogs of the essential metabolites to inhibit enzymes that relied on them as substrates (59).

Constraint-based modeling methods have also been used to model interactions between different cell types (70). Following a similar workflow, it is possible to use GEMs of the cancerous and non-cancerous cells in a tumor microenvironment to identify essential metabolites whose inhibition would disrupt the symbiotic relationship between cancerous cells and non-cancerous cells in their surroundings. For instance, recent data have indicated that glycolysis-targeting interventions such as the depletion of PFKFB3 may exert antineoplastic effects by limiting vessel sprouting (27), hence targeting both proliferative endothelial and cancer cells. Thus, outlining the common metabolic features between normal and pathological cells can be of potential clinical value.

Constraint-based modeling allows prediction of numerous metabolic phenotypes, including growth rates, nutrient uptake rates, and gene essentiality. They are, thus, well-suited to the search for common metabolic features across a span of pathological and physiological conditions as well as for integration in the early stages of target-based drug development pipelines.

## Concluding Remarks

Although WE is one of the most extensively studied bioenergetic processes that are being shared between cells that undergo rapid proliferation, other bioenergetic and anabolic processes contain similar potential to being metabolic phenotypes of rapid proliferation. For instance, glutamine dependency and glutaminolysis increased PPP activity, serine and glycine metabolism as well as *de novo* lipogenesis (71, 72). Likewise, several intermediate metabolites bear the potential of being biomarkers of rapid proliferation (e.g., serine, sarcosine, and kyneurin). However, whether a therapeutic window for the clinical application of these processes exists remains to be determined. Noteworthy is that the response of the glycolytic pathway to drug perturbations is non-linear (71–73). Thus, careful considerations will be needed to develop a biomarker that can determine the context in which it would be efficacious to exploit any diagnostic or therapeutic potential for the WE. The clinical success of antimetabolites (71) lends support to the argument presented here that metabolic events can be therapeutically exploited while being shared between both normal and pathologic rapidly proliferating cells. Nevertheless, drug inhibitors developed against other metabolic events have not progressed beyond the pre-clinical stages yet (74, 75). Taken together, the arguments and discussion presented here suggest that grouping diseases and cell types according to common metabolic phenotypes can provide mechanistic understanding of the observed phenotypes in relation to the context-specific repertoire of metabolic interactions as well as expediting drug development pipelines.

## Author Contributions

AMA and TG conceived and synthesized the study. The manuscript was written by AMA and TG with input from NL, NJ, KM and XG.

## Conflict of Interest Statement

The authors declare that the research was conducted in the absence of any commercial or financial relationships that could be construed as a potential conflict of interest.
